# RNA-seq analysis identified glucose-responsive genes and YqfO as a global regulator in *Bacillus subtilis*

**DOI:** 10.1186/s13104-021-05869-1

**Published:** 2021-12-14

**Authors:** Yu Kanesaki, Mitsuo Ogura

**Affiliations:** 1 Research Institute of Green Science and Technology, Shizuoka University, 836 Ohya, Suruga-ku, Shizuoka, 422-8529 Japan; 2 Institute of Oceanic Research and Development, Tokai University, 3-20-1 Orido Shimizu-ku, Shizuoka, 424-8610 Japan

**Keywords:** RNA-seq, Glucose, Catabolite regulation, Transcriptome

## Abstract

**Objective:**

We observed that the addition of glucose enhanced the expression of *sigX* and *sigM,* encoding extra-cytoplasmic function sigma factors in *Bacillus subtilis*. Several regulatory factors were identified for this phenomenon, including YqfO, CshA (RNA helicase), and YlxR (nucleoid-associated protein). Subsequently, the relationships among these regulators were analyzed. Among them, YqfO is conserved in many bacterial genomes and may function as a metal ion insertase or metal chaperone, but has been poorly characterized. Thus, to further characterize YqfO, we performed RNA sequencing (RNA-seq) analysis of YqfO in addition to CshA and YlxR.

**Results:**

We first performed comparative RNA-seq to detect the glucose-responsive genes. Next, to determine the regulatory effects of YqfO in addition to CshA and YlxR, three pairs of comparative RNA-seq analyses were performed (*yqfO*/wt, *cshA*/wt, and *ylxR*/wt). We observed relatively large regulons (approximately 420, 780, and 180 for YqfO, CshA, and YlxR, respectively) and significant overlaps, indicating close relationships among the three regulators. This study is the first to reveal that YqfO functions as a global regulator in *B. subtilis*.

**Supplementary Information:**

The online version contains supplementary material available at 10.1186/s13104-021-05869-1.

## Introduction

Glucose is used as the most favorable carbon source for many gram-positive bacteria. Hence, bacteria have developed a variety of glucose-responsive systems. For example, the gram-positive model bacterium *Bacillus subtilis* possesses the transcription factor catabolite control protein A (CcpA) as the primary carbon catabolite regulator [1, 2]. CcpA causes global transcriptional changes, and additional glucose-responsive transcription factor genes are detected in the *B. subtilis* genome [2]. Several DNA microarray analyses, however, have revealed many glucose-responsive genes in which glucose responses are caused by unknown factors [3–5].

**Fig. 1:**
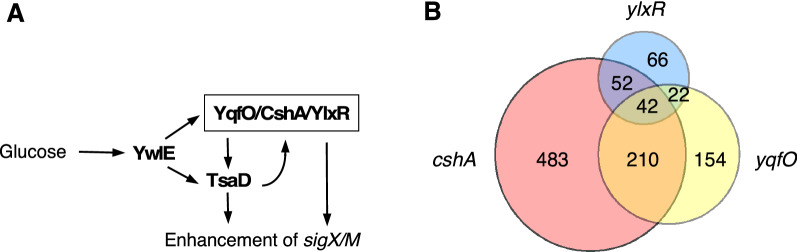
Glucose induction of *sigX/M* and Venn diagram of the detected regulons. **A** Arrows indicate positive regulation and do not mean direct effects. Details are in the Refs. [16, 21]. **B** Numbers indicate gene numbers in the sections

We observed that glucose in the medium enhanced the expression of *sigX* and *sigM,* encoding extra-cytoplasmic function sigma factors [6] (Fig. 1A). We identified several regulatory factors for this phenomenon, including YqfO, CshA, and YlxR, and analyzed the relationships among these regulators [7, 8]. YqfO is a conserved protein among the bacteria of the Firmicutes phylum that bears a DUF34/nif3 conserved domain with a suggested function related to transcriptional regulation [9]. The structure of *Bacillus cereus* YqfO has been resolved, revealing the presence of a dimetal-binding motif [10]. Recently, bioinformatics analysis using data from the determined genome sequences and published reports revealed that YqfO may function as a metal chaperone or metal insertase [11]. We observed that YqfO is under positive control of YlxR [8].

CshA, a DEAD-box helicase associates with RNA polymerase (RNAP) [12, 13]. RNAP associated with CshA is involved in the glucose induction (GI) of *ylxR* [6]. YlxR is a nucleoid-associated protein and regulates the expression of several hundred genes, including *tsaD* [7]. TsaD is a component of the tRNA modification enzyme TsaEBD, which is thought to be involved in protein translation [14]. TsaD stability is also controlled by the GI of protein arginine phosphatase YwlE, because protein arginine phosphorylation leads to ClpCP-dependent protein degradation [15, 16]. Additionally, the expression of an operon containing a gene encoding protein arginine phosphorylase McsB is under catabolite repression [17]. As shown in Fig. 1A, these regulators are in the feedback regulatory loop, and perhaps CshA-associated RNAP is directly involved in the GI of *sigX/M*. Based on the previous and RNA-seq analyses, close relationships among these regulatory factors for GI were identified. The RNA-seq analysis of YqfO revealed that YqfO functions as a global regulator in *B. subtilis* and confirmed that YqfO is involved in the regulatory network for GI composed of CshA, YlxR, and TsaD.

## Main text

### Methods

#### Strains and media

The wild-type *B. subtilis* strain 168 was used for identification of glucose-responsive genes. The strain was grown in 50 ml of the sporulation medium (SM) with or without 2% glucose. For RNA-seq of three regulatory factors, the wild-type (OAM829 bearing *amyE*::*trmK-lacZ*) and its derivatives, OAM953 (*cshA*::Tc^r^, this study), OAM829 (*ylxR*::pMUTIN-*lacZ*::Tc^r^) [7], and OAM954 (*yqfO*::pMUTIN-*lacZ*::Tc^r^, this study) were used. OAM950 (*pftA*::pMUTIN-pftA[Em^r^], this study) and its derivatives OAM951 (*yqfO*[Em^r^, *lacZ*::Tc^r^], this study) and OAM952 (*cshA*[Tc^r^], this study) were constructed as follows. pMUTIN-pftA was constructed by insertion of the PCR product amplified by pMut-pftA-H(5-ATCAAGCTTATGCCGGAACAGAAGATG-3) and pMut-pftA-B(5-ATGGGATCCTTTCTTCACCTCTTTCTCTTTGG-3) after digestion with HindIII and BamHI to pMUTIN treated with the same enzyme pair [8]. OAM955 (*fruR*::pMUTIN-fruR [Em^r^], this study) and its derivative OAM956 (*yqfO*[Em^r^, *lacZ*::Tc^r^], this study) were constructed as follows. pMUTIN-fruR was constructed by insertion of the PCR product amplified by pIS-fruR-E (5-ATCGAATTCCTGGCAGGTTGTATGC-3) and pMut-fruR-BB (5-ATGGGATCCACCATGAACGCGCTTT-3) after digestion with EcoRI and BamHI into pMUTIN treated with the same enzyme pair [8]. The resultant plasmids were used for genetic transformation to generate OAM950 and OAM955, and then each gene disruption was introduced in OAM950 or OAM955.

#### RNA isolation and RNA-seq analysis

For the experiments shown in Additional file 2: Table S1, 168 was grown in 50 ml of SM with or without 2% glucose and cell culture was sampled at T2 (2 h after the end of the logarithmic growth phase). For the experiments shown in Additional file 2: Tables S2–4, each strain was grown in 50 ml of SM with 2% glucose. Cell culture was sampled at T2. RNA isolation was performed as previously described [7]. Briefly, RNA was isolated from the cells collected by centrifugation using an RNeasy Mini Kit (Qiagen, Germantown, MD, USA). RNA-seq was performed as described in Additional file 1: Supplementary Methods.

#### β-Gal analysis

β-Gal analysis was performed as described previously [6].

#### Results and discussion

**Fig. 2:**
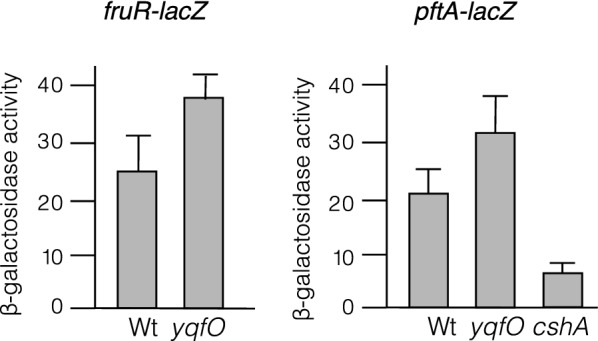
Expression of P*fruR-lacZ* and P*pftA-lacZ* in SM with 2% glucose in early stationary phase (T1). Y-axis shows β-Gal activities in Miller units. T-bar shows standard deviation derived from three independent experiments

YqfO, CshA, and YlxR were identified as the controlling factors involved in GI of *sigX/M* in the early stationary phase cells in a sporulation medium (SM) with 2% glucose [6, 8]. Hence, we first performed comparable RNA-sequencing (RNA-seq) of wild-type *B. subtilis* 168 cells in SM with or without 2% glucose to detect glucose-responsive genes. We identified 528 (threshold  × 10) and 1494 (threshold  × 3) of the upshift and downshift genes with glucose, respectively (Additional file 2: Table. S1a, b). This showed the powerful detectability of differentially expressed genes (DEGs) by RNA-seq, because the former analyses using DNA microarray detected 852 (threshold  × 2) [3], less than 100 (threshold  × 3) [4], and 503 (threshold  × 3) [5] DEGs related to glucose addition. CshA- and YlxR-regulated genes have been identified by DNA microarray and RNA-seq, respectively, and are approximately 200 and 400 genes, respectively [7, 13], whereas YqfO-regulated genes have not been characterized. Thus, we performed comparable RNA-seq of the wild-type and its derivatives bearing the disruption of *yqfO, cshA*, and *ylxR*. In Additional file 2: Tables S2–4, each DEG (428, 787, and 182) detected by RNA-seq analysis is shown. As a result, we first observed that disruption of the *yqfO* gene has a broad impact on genome gene expression. YqfO belongs to a large protein superfamily with unknown functions (DUF34), which is conserved in all three domains of life [11]. Although an exact mechanistic analysis was lacking, pleiotropic effects on physiological aspects, including transcription regulation, were observed in the disruptants of the genes encoding DUF34 proteins in many organisms [11]. The *Thermus thermophilus* DUF34 protein YbgI binds to single-stranded DNA [18], and the *Geobacillus stearotherophilus* DUF34 protein XynX regulates the *xynA* gene encoding xylanase through its binding to the *xynA* promoter [19]. Our RNA-seq analysis for YqfO also revealed more than 400 genes that are under the control of YqfO, suggesting the pleiotropic functions of YqfO in *B. subtilis*. Notably, the previous study on YqfO revealed only one transcription unit under the control of YqfO [8]. The expression of one of the target operons in RNA-seq, *fruRKA* (fructose metabolic operon), was confirmed to be affected by *yqfO* disruption using β-Gal analysis (Fig. 2). *B. subtilis* YqfO affects the transcription of many genes through unknown mechanisms. It is possible that YqfO may do so through regulation of metal ion homeostasis as the metal chaperone or metal ion insertase, which affects many enzyme activities, because YqfO-His did not bind to the target promoter region in the electromobility shift assay (Additional file 1: Supplementary Methods; Additional file 3: Fig. S1).

Compared to the previous report [7], the number of YlxR-regulated genes in this study was relatively small, perhaps because of the different algorithms for DEGs. Compared to the inventory of the glucose-regulated genes, the detected CshA-regulon did not show a particular bias for that inventory (Additional file 2: Tables S1, S3), indicating that many CshA-regulon genes may play other roles in glucose response. It should be noted that the previous studies revealed extreme upregulation of the *pftAB* (*ysbAB*, encoding pyruvate transporter) [20] and downregulation of the *frl* (encoding proteins involved in fructoselysine utilization) operons in the *cshA* disruptant [13]. Our study detected upregulation of *pftAB* in the *yqfO*-disruptant but not in the *cshA*-disruptant. β-Gal analysis of *pftA* expression in the *yqfO* disruptant confirmed our RNA-seq results (Fig. 2). Moreover, we observed downregulation of *pftA* in the *cshA* disruptant, which is contrary to the previous results; the reason behind this is unknown. It should be noted that our β-Gal experiments were performed with glucose and different media whereas previous experiments involved the use of LB medium. We observed differential expression of the *frl* operon among all three disruptants analyzed, especially downregulation in the *cshA* disruptant (Table 1B). A detailed analysis of the *frl* operon expression in the *ylxR* disruptant has been described previously [21]. A previous study indicated a regulatory network including CshA/YlxR/YqfO (Fig. 1A) [6–8]. Overlapping genes in the three regulons are shown in Fig. 1B as Venn diagrams and in Additional file 2: Tables, S2–4. Figure 1B shows that 52% of the YlxR- and 59% of the YqfO-regulon genes overlapped with the CshA-regulon genes, confirming close relationships among the three regulators. A certain group of genes whose expression was independent of YlxR or YqfO was observed in the CshA-regulon. This may be related to the former observation that CshA is a component of RNA degradosome including RNase Y, which controls mRNA abundance of at least 10% of the genes in the genome [13]. We note that in most of the genes regulated by CshA and YqfO, gene disruption effects have the same direction; that is, when *cshA* disruption affected the expression of particular genes positively, *yqfO* disruption also did so (see Additional file 2: Table S2). Moreover, it should be noted that this estimation of overlap might be underestimated, because all the genes contained in the specific operon were not always detected in the RNA-seq analysis, as shown in Table 1. These operons in Table 1 encode metabolic genes involved in the biosynthesis of histidine and pyrimidine, and degradation of fructoselysine. The genes commonly detected in the three regulons are listed in Additional file 2: Table S5 and include several metabolic genes, for example, *bglH* (aryl-phospho-beta-D-glucosidase), *ctaD/E/G* (cytochrome c oxidase subunits/ assembly factor), *rocE/rocF* (amino acid permease/arginase), *manA* (mannose-6-phosphate isomerase), and *mtlD* (mannitol-1-phosphate 5-dehydrogenase). The RNA-seq analyses of the three regulons confirmed close relationships with each other, suggesting the reliability of the obtained RNA-seq results. Moreover, Venn diagram shows that YlxR and YqfO also have their own regulon genes that are not under the control of other regulators. These results suggest that YlxR and YqfO have distinct roles other than GI of the genes. Hence, these RNA-seq results should be useful for the further research.

**Table 1:** Each gene expression of *cshA/ylxR/yqfO*-regulated operons

A. *his* operon	*hisZ*	*hisG*	*hisD*	*hisB*	*hisH*	*hisA*	*hisF*	*hisI*		
*cshA*	R	R	R	R	R	R	R	R		
*ylxR*	R	R	R	R	R					
*yqfO*		R	R	R	R					
Glucose	E	E	E	E	E	E	E	E		
B. *frl* operon	*frlB*	*frlO*	*frlN*	*frlM*	*frlD*	*yurJ*				
*cshA*	R									
*ylxR*	E	E	E	E	E	E				
*yqfO*	R	R								
Glucose	R	R	R	R	R	R				
C. *pyr* operon	*pyrR*	*pyrP*	*pyrB*	*pyrC*	*pyrAA*	*pyrAB*	*pyrK*	*pyrD*	*pyrF*	*pyrE*
*cshA*	E			R	R		R	R	R	R
*ylxR*			R	R	R		R	R	R	R
*yqfO*							R			
Glucose	E	E	E	E	E	E	E	E	E	E

CcpA-dependent glucose-activation of the *pyr* operon was reported in Moreno et al. [3]

*R *repressed in the gene-disruptant or with glucose; *E *enhanced in the gene-disruptant or with glucose

Operon structure; see Subtiwiki (http://subtiwiki.uni-goettingen.de/)

## Limitations

In this study, RNA-seq analysis was performed once per pair for comparison (N  = 1); hence the inventory of the genes with marginal fold-change may change if additional RNA-seq experiments are carried out. However, many of the gene lists with critical fold-change will not change, thus, the information in this study would be valuable for researchers studying gene expression in *B. subtilis.*

## Supplementary Information

**Additional file 1: **Supplementary methods.

**Additional file 2: Table S1.** a Glucose-regulated genes (high threshold). b Glucose-regulated genes (low threshold). **Table S2.** YqfO-regulated genes. **Table S3.** CshA-regulated genes. **Table S4.** YlxR-regulated genes. **Table S5.** *cshA/ylxR/yqfO*-regulated genes.

**Additional file 3: Figure S1.** Electromobility shift assay of YqfO using the promoter region of the *thiL* operon.

## Data Availability

The data underlying this article are available in the Sequence Read Archive at https://www.ddbj.nig.ac.jp/dra/index.html. The data for wild-type samples without or with glucose can be accessed with DRR296186 and DRR296187, respectively. The other data of the samples with *amyE*::*trmK-lacZ* are as follows; Wt, DRR139003 [W1.fq.gz, W2.fq.gz]; *cshA*, DRR296184; *ylxR,* DRR139004 [R1.fq.gz, R2.fq.gz]; and *yqfO*, DRR296185. All other data generated or analyzed during this study are included in this published article and its Additional files.

## Abbreviations

RNA-seq: RNA sequencing
SM: Sporulation medium
GI: Glucose induction
RNAP: RNA polymerase
DEAD box: Asp-Glu-Ala-Asp box
PCR: Polymerase chain reaction
DEG: Differentially expressed gene
