# Supplementary material for: RNA-seq analysis identified glucose-responsive genes and YqfO as a global regulator in Bacillus subtilis
Source: BMC Res Notes. 2021 Dec 14;14:450. doi: 10.1186/s13104-021-05869-1 (PMC8670212; doi:10.1186/s13104-021-05869-1)
Supplement: Supplementary file 1 — Additional file 1: Supplementary methods. [file 13104_2021_5869_MOESM1_ESM.docx]

**RNA-seq analysis identified glucose-responsive genes and YqfO as a global regulator in *Bacillus subtilis***

Yu Kanesaki and Mitsuo Ogura

**Supplementary Method**

**RNA-seq analysis**

Libraries for RNA-sequencing were constructed using 1 micro-g of total RNA as follows. rRNA was depleted from total RNA by the Ribo-Zero rRNA Removal kit (Bacteria) (Illumina) according to the manufacturer’s protocol. Sequencing libraries were prepared by NEBNext Ultra II Directional RNA Library Prep Kit for Illumina (NEB) with the rRNA depletion workflow of the manufacturer’s protocol. 76 base pairs from both ends of each fragment were then sequenced on the MiSeq system platform (Illumina). After the sequencing reactions were complete, the MiSeq Reporter was used to process the raw sequencing data. The reads were trimmed using CLC Genomics Workbench ver. 11.0 with the following parameters; Phred quality score > 30; ambiguous nucleotides allowed: 0; automatic read-through adaptor trimming: yes; removing the terminal 15 nucleotides from the 5' end and 2 nucleotides from the 3' end; and removing truncated reads of less than 30 nucleotides in length. Trimmed reads were mapped to the all genes in Bacillus subtilis str.168 (accession number: AL009126.3) using CLC Genomics Workbench ver. 11.0 (Qiagen) with the following parameters; match score: 1; mismatch cost: 2; indel cost: 3; length fraction: 0.7; similarity fraction: 0.9; and maximum number of hits for a read: 1. Original read count of each gene was normalized with total read counts as one million after adding value of 2 to the raw counts to avoid zero read counts. For the identification of differentially expressed genes between the WT and the mutant cells, proportion-based statistical analysis algorithm using Baggerley’s test with FDR corrected p-value in the RNA-seq module in CLC Genomics Workbench ver. 11.0 was applied.

RNA-Seq in Tables S2, S3, and S4 was carried out by Novogene, Inc. (Hong Kong) and the Rpackage DEGseq was used for data analysis.

**Plasmid construction.**

The plasmid pET28-yqfO was constructed by cloning of the PCR products amplified by using the oligonucleotide pair yqfO-Sc(pET) (CTAGAGCTCATGGCTAAAAGTGTAAATGGGC) and yqfO-chitin-R (Xh) (TTGCTCGAGTTATAGAAATGTAAATGGATTTGTATC) and digested with SacI and XhoI into the pET28a plasmid treated with the same enzyme pair (Qiagen).

**Purification of YqfO-His**

The *E. coli* strain BL21(DE3) bearing pET28-yqfO was grown in 600 ml of LB medium (20 μg/ml kanamycin) at 30˚C. At an OD600 of around 0.8, 0.2 mM IPTG was added, and the culture was further incubated for 20 h at 20°C. After harvest of the culture, the cells were processed and YqfO-His was purified using a Ni-affinity column as described previously [1]. After SDS-PAGE analysis of the fractions, the purified protein was dialyzed against buffer containing 10 mM Tris-HCl (pH 8.0), 100 mM KCl, 10 mM MgCl2, 1 mM DTT. Aliquots of the purified proteins were stored at -80˚C.

**Electromobility shift assay**

The oligonucleotides used for preparing probe are gcp-biotin-R (biotin- GAACCAAAACCTCCTTTCAACC) and pDG1729-gcp-E2 (ATTGAATTCGTGCCCTAAGGGTGCAACCA). The amplified region from *B. subtilis*168 total DNA contains the cis-element for YqfO-regulation [2]. His-tagged YqfO was incubated with each DNA probe (20 fmol) in 20 ml of a buffer containing 10 mM Tris-HCl (pH 8.0), 100 mM KCl, 10 mM MgCl2, 1 mM DTT, 1mM EDTA for 15 min at 25˚C. After the addition of 2 μl of a loading buffer (40 % glycerol, 1 x TBE, and 2 mg/ml bromophenol blue), the samples were applied onto a 6% non-denaturing polyacrylamide gel and electrophoresis was performed in 0.1 x TBE buffer at 4˚C. The detection of biotin-labelled DNA was described previously [1].

[1] Tsukahara K, Ogura M. BMC Microbiol 2008;8:8.

[2] Ogura M, Asai K. Front Microbiol 2016;7:1918.
