# Supplementary figures and images for: RNA-seq analysis identified glucose-responsive genes and YqfO as a global regulator in Bacillus subtilis
Source: BMC Res Notes. 2021 Dec 14;14:450. doi: 10.1186/s13104-021-05869-1 (PMC8670212; doi:10.1186/s13104-021-05869-1)

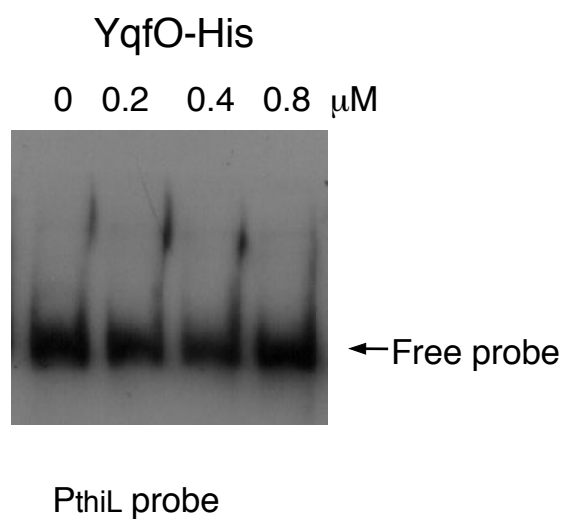

Fig. S1

Supplement: Supplementary file 3 — Additional file 3: Figure S1. Electromobility shift assay of YqfO using the promoter region of the thiL operon. [file 13104_2021_5869_MOESM3_ESM.pdf]
